# Overall outcome, functioning, and disability in older adults 3 to 14 years after traumatic brain injury

**DOI:** 10.1002/pmrj.70012

**Published:** 2025-09-19

**Authors:** Charlotta von Seth, Anders Lewén, Marianne Lannsjö, Per Enblad, Jan Lexell

**Affiliations:** ^1^ Department of Medical Sciences Uppsala University Uppsala Sweden; ^2^ Uppsala University Hospital Uppsala Sweden; ^3^ Department of Health Sciences Lund University Lund Sweden

## Abstract

**Background:**

Epidemiological studies show an increasing incidence of traumatic brain injury (TBI) among people aged 65 years and older. Advances in neurointensive care have improved survival after TBI. There is a need for knowledge about long‐term outcome after TBI among older survivors of TBI.

**Objective:**

To describe the overall outcome, long‐term functioning, and disability of the participants in the Uppsala Long‐term outcome in Older adults with Traumatic brain injury Study (U‐LOTS), a cohort study assessing adults aged 60 years or older when admitted to a neurointensive care unit following a TBI, where 3 to 14 years had passed since the injury.

**Design:**

Cross‐sectional cohort study.

**Setting:**

Home and community settings.

**Interventions:**

Not applicable.

**Participants:**

Data were collected from 79 survivors of TBI (65% men; mean age 76 years, mean time since TBI 7 years).

**Main Outcome Measures:**

The Glasgow Outcome Scale Extended (GOSE), the Functional Independence Measure (FIM), the Mayo‐Portland Adaptability‐4 (MPAI‐4), and the following sociodemographics and TBI characteristics: gender, age, marital status, vocational situation, rehabilitation, need of assistance, use of mobility devices, oropharyngeal dysphagia, impaired sense of smell and/or taste, prior brain disease, cause of accident, severity of TBI, comorbidities, dominant finding on first computed tomography scan, and assessment with GOSE 6 months after TBI.

**Results:**

Falls (68%) and acute subdural hematoma (41%) were the most common cause and injury. For many participants (40%) the GOSE scores did not change between 6 months after TBI until the long‐term follow‐up, and a majority (57%) had a relatively good outcome as assessed with the MPAI‐4. GOSE, FIM, and MPAI‐4 Ability scores were significantly (*p* < .05) correlated with injury severity. Marital status remained unchanged for 70% of the participants.

**Conclusions:**

Older adults surviving a TBI may have a relatively favorable outcome. A major factor that determined long‐term outcome was injury severity.

## INTRODUCTION

Traumatic brain injury (TBI) is one of the leading causes of death and disability worldwide,^1^, ^2^ and every year 69 million individuals of all ages are estimated to sustain a TBI.^1^ The clinical outcome has improved as a result of improved neurointensive care (NIC), but many people still experience remaining lifelong physical, cognitive, and emotional impairments that can lead to activity limitations and participation restrictions.^3^ Researchers have considered TBI as a chronic condition where functioning and health may fluctuate, and often deteriorate over time. Neurodegenerative processes, comorbidities, social determinants of health, and increasing age are some factors that may contribute to adverse outcomes.^4^, ^5^, ^6^

Epidemiological studies report a rise in TBI among people aged 65 years and older.^3^, ^7^, ^8^, ^9^ This is in line with the ongoing worldwide demographic shift toward an aging population.^10^ Several studies have shown that older people with TBI have higher mortality rates and are less likely to function independently after TBI compared to younger people.^3^, ^11^, ^12^ Older people are also more likely to have preexisting medical conditions that can impede their recovery and rehabilitation.^6^, ^8^, ^11^, ^13^

Studies of older people with TBI who receive modern NIC have shown that they can, in fact, have a relatively good clinical outcome.^14^, ^15^, ^16^, ^17^, ^18^ However, most clinical studies of remaining disability have generally been performed in the early phase, often within the first year post injury,^19^, ^20^, ^21^ despite our knowledge that outcomes can both improve or worsen many years post injury.^22^ Assessing outcome of TBI is complex, as a variety of factors in different domains and over time may contribute to improvement or deterioration.^4^ For a better understanding of the long‐term consequences of TBI, it is therefore important to evaluate outcomes over a longer period post injury including not only injury severity, but also various factors influencing functioning, disability and health.

To contribute to our knowledge about long‐term outcome after TBI among older people, the Uppsala Long‐term outcome in Older adults with Traumatic brain injury Study (U‐LOTS) was initiated. The U‐LOTS is a cohort study spanning an 11‐year period assessing adults with TBI with mild, moderate, and severe injury who were 60 years or older at the time of injury and admitted to a NIC unit. The overarching aim of U‐LOTS is to describe overall outcome, long‐term functioning, and disability, as well as life satisfaction and personal and environmental factors in these people 3 years or more post injury and identify factors that may affect long‐term outcome.

The specific aim of the present study is to describe the overall outcome, long‐term functioning, and disability of the participants in U‐LOTS.

## MATERIALS AND METHODS

### Study design

The U‐LOTS is a cross‐sectional cohort study targeting older adults who sustained a TBI and were admitted to the NIC unit at Uppsala University Hospital (catchment area of approximately 2 million inhabitants from the middle part of Sweden). All participants in the U‐LOTS were recruited from the Uppsala Traumatic Brain Injury (UTBI) register.^23^, ^24^ The UTBI register was established in 2008 and includes all patients aged 16 years or older who are admitted to the NIC unit at Uppsala University Hospital, Sweden, following a TBI. The UTBI register provides background data from the time of the injury as well as results of the assessment with the Glasgow Outcome Scale Extended (GOSE)^25^, ^26^, ^27^, ^28^ around 6 months post injury. Potential participants in the U‐LOTS were those in the UTBI register who were 60 years or older at the time of TBI and were at least 3 years post injury.

### Ethical considerations

The U‐LOTS was approved by the Regional Ethical Review Board in Uppsala, Sweden (No. 2018/452) and the Declaration of Helsinki for research on humans was followed. Before enrolment and signing an informed consent, the participants were given written information about the study and that they could withdraw at any time.

### Study population

**FIGURE 1:**
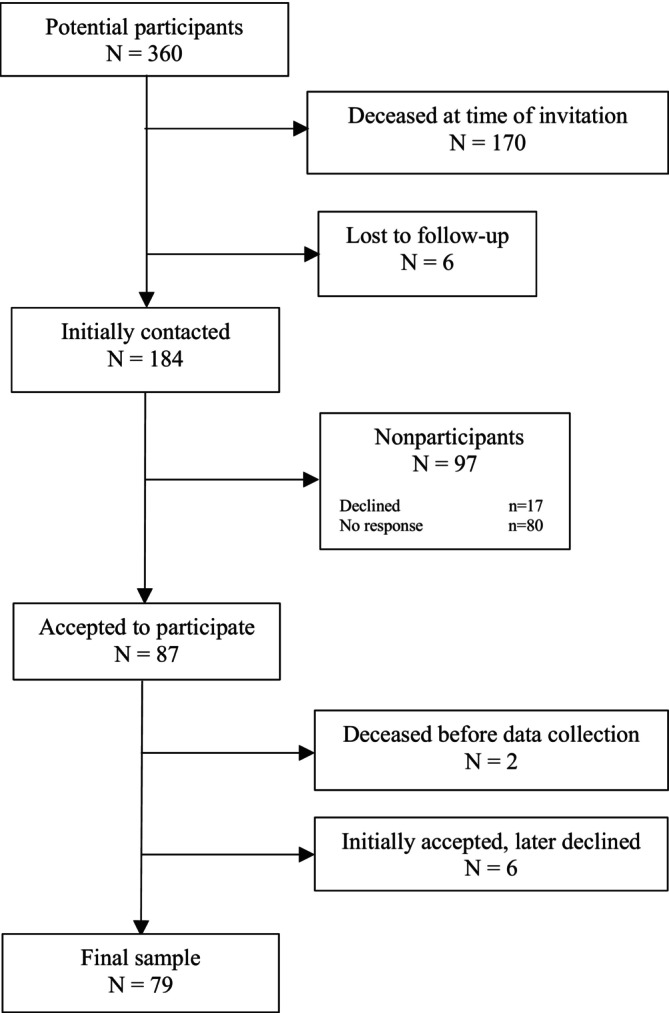
The recruitment procedure of the Uppsala Long‐term outcome in Older adults with Traumatic brain injury Study (U‐LOTS).

From the UTBI register all patients 60 years or older who had been admitted to the NIC unit between the years 2008 and 2019 were identified as potential participants. At the start of the U‐LOTS, the register comprised 360 potential participants (Figure 1). At the time of invitation to participate in the study, 170 of the potential participants were deceased. Another six were lost to follow‐up because of only temporary Swedish social security number or no registered address. A total of 184 men and women matched the inclusion criteria and were invited to participate. They were contacted by letter with information about the study and an informed consent form. One reminder was sent to those who did not respond after the first invitation, and if still no response, attempts to establish contact via telephone were made. Of the 184 potential participants, 87 accepted the invitation, 17 declined and 80 did not respond despite several attempts to make contact. Six of those who initially accepted, later declined participation, and two were deceased before the data collection commenced. The final sample comprised 79 participants.

### Data obtained from the UTBI register

From the UTBI register, the following data were obtained: prior brain disease, diabetes mellitus, cardiovascular diseases, anticoagulation treatment, cause of accident, injury severity on admission to the NIC unit (assessed with the Reaction Level Scale [RLS 85]^29^ and the Glasgow Coma Scale‐Motor [GCS‐M]^30^), dominant findings on the first computed tomography scan, neurological status at discharge from the NIC unit (RLS 85 and GCS‐M), and evaluation of outcome using the GOSE^25^, ^26^, ^27^, ^28^ 6 months after discharge from the NIC. The RLS 85 scores were transformed into GCS scores and based on these scores the participants were divided into the three common TBI severity groups: mild TBI = RLS 1‐3a/GCS 13‐15; moderate TBI = RLS 3b‐4/GCS 9‐12; and severe TBI = RLS 5‐8/GCS 3‐8.^31^, ^32^, ^33^

### Data collection at the long‐term follow‐up

All long‐term follow‐up data, including all outcome measures, were collected by the first author between December 2018 and September 2023. Data were obtained through structured interviews with a study‐specific protocol and internationally established TBI outcome measures either during visits (*n* = 73) or via telephone (*n* = 6), and lasted on average 120 minutes (100–220 minutes). The following data from the study‐specific protocol were obtained: rehabilitation (provided by the municipality/primary health care and/or hospital/community based rehabilitation services), need of assistance (including personal assistance, home‐help service provided by the municipality, dependent on next‐of‐kin/significant other, help with cleaning/household/maintenance by private home care service or a combination of these), use of walking device, use of wheelchair, oropharyngeal dysphagia, impaired sense of smell and/or taste, marital status at the time of injury and at follow‐up (recorded as living alone, cohabitant or living‐apart together relationship), and vocational situation at follow‐up (recorded as old age retirement or working full time/part time).

### Data obtained with the outcome measures

#### Glasgow Outcome Scale Extended

The GOSE is an extended version of the GOS to classify global outcome after TBI.^25^, ^28^ In 1998 a structured interview questionnaire was introduced to standardize procedures for scoring both GOS and GOSE aiming to improve the reliability of ratings.^26^, ^27^ The interview contains a set of guiding questions about injury‐related deterioration in seven categories: ability to take part in daily life, work, social activities, and family relations. Outcome is graded 1 to 8 (1 = dead; 8 = upper good recovery) and the level of recovery depends on the answers in the different categories instead of a sum score of individual items. The GOSE interview also includes questions about epilepsy following head injury and which factor (effects of head injury or effects of illness or injury to another part of the body or a mixture of these) is the most important in outcome.

#### Functional Independence Measure

The Functional Independence Measure (FIM) assesses the degree of assistance required to perform personal activities of daily living.^34^ It consists of 18 items separated into two domains: the motor domain (*n* = 13) and the cognitive domain (*n* = 5). Each item is scored on a seven‐level ordinal scale; a score of 1 indicates need of total assistance and a score of 7 complete independence. In the motor domain, a sum score of ≥78 (total score 91) and in the cognitive domain a sum score of ≥30 (total score 35) indicates complete or modified independence.

#### Mayo Portland Adaptability Inventory

The Mayo‐Portland Adaptability‐4 (MPAI‐4) is an outcome measure consisting of 35 items that are scored on a 5‐point Likert scale.^35^ The items represent physical, cognitive, emotional, behavioral, and social problems which are frequent sequelae of TBI.^36^ Twenty‐nine of the items are within three subscales and an overall score: Ability Index (range 0–51), Adjustment Index (range 0–46), Participation Index (range 0–30), and an overall score of 0–115 (Raw score), with lower scores indicating greater integration (ie, better outcome). Three of the items are part of both the Adjustment and the Participation subscale scores; this explains why the overall score is less than the sum of the three subscale scores. The additional 6 items are nonscored and address factors that may contribute to the functional status. For participants considered in a minimally conscious state, the MPAI‐4 was administered in a nonstandard manner according to the developer of MPAI‐4, and 4 was given on items to which they could not respond (James F. Malec, personal communication). Raw scores from the Total score and the three subscales of the MPAI‐4 were converted to standardized T‐scores by using tables based on the 2015 National OutcomeInfo Sample as reference.^37^ According to the recommendation in the MPAI‐4 manual,^38^ total T‐scores <30 represent relatively good outcomes, 30–40 suggest mild limitations, 40–50 are considered in the mild to moderate range, 50–60 in the moderate to severe range of overall severity compared to most other people with acquired brain injury, and T‐scores >60 indicate severe limitations.^38^

### Data and statistical analysis

Data are presented as mean, SD, minimum and maximum, and median and interquartile range, where appropriate. Differences between participants and nonparticipants were analyzed with the Mann–Whitney *U* test or the Pearson chi^2^ test. Relationships between variables were analyzed with the Spearman rank correlation coefficient. All statistical analyses were performed using the IBM SPSS version 28.0.1.0 (IBM Corporation, Armonk, NY). *P* values < .05 represent statistical significance.

## RESULTS

### Participants and nonparticipants

Data on the 79 participants and the 105 nonparticipants are presented in Table 1. In both groups men were in the majority and ≥50% had sustained a mild TBI. There were no significant differences between the participants and the nonparticipants regarding gender (*p* = .204), age at injury (*p* = .514), or injury severity (*p* = .278).

**TABLE 1:** Comparison between the participants and nonparticipants in the Uppsala Long‐term outcome in Older adults with Traumatic brain injury Study (U‐LOTS).

	Participants	Nonparticipants	Significance
	(*n* = 79)	(*n* = 105)	
Gender			
Male, *N* (%)	51 (65)	80 (76)	*p* = .204^b^
Female, *N* (%)	28 (35)	25 (24)	
Age at injury, mean ±SD, median [min‐max], (IQR)	69 ± 6, 68 [60–84], (64–72)	68 ± 6, 67 [60–87], (64–72)	*p* = .514^c^
Injury severity^a^			
Mild TBI, *N* (%)	44 (56)	52 (50)	*p* = .278^c^
Moderate TBI, *N* (%)	29 (37)	37 (35)	
Severe TBI, *N* (%)	6 (8)	16 (15)	

Abbreviations: IQR, interquartile range (25th‐75th percentile); RLS 85, Reaction Level Scale; TBI, traumatic brain injury.

^a^ Injury severity was classified as level of consciousness at the injury: Mild TBI = RLS 85 1‐3a; Moderate TBI = RLS 85 3b‐4; Severe TBI = RLS 85 5‐8.

^b^ Differences in frequencies between participants and nonparticipants were tested with Pearson chi^2^ test.

^c^ Differences between participants and nonparticipants were tested with Mann–Whitney *U* test.

### Descriptive findings for the participants

Data on injury characteristics and sociodemographics at the long‐term follow‐up for the 79 participants are presented in Table 2. A majority of the participants (*n* = 51; 65%) were men.

**TABLE 2:** Injury characteristics and sociodemographics at the long‐term follow‐up for the 79 participants in the Uppsala Long‐term outcome in Older adults with Traumatic brain injury Study (U‐LOTS)

	Total group	Mild TBI^a^	Moderate TBI^a^	Severe TBI^a^
	(*n* = 79)	(*n* = 44)	(*n* = 29)	(*n* = 6)
Gender, *n* (%)				
Men	51 (65)	29 (66)	19 (65)	3 (50)
Women	28 (35)	15 (34)	10 (35)	3 (50)
Age at long‐term follow‐up, mean ±SD, median, min‐max	76 ± 5, 75, 64‐91	76 ± 5, 76, 67‐90	75 ± 5, 73, 67‐91	76 ± 7, 78, 64‐83
Time since injury (years), mean ±SD, median, min‐max	7 ± 3, 7, 3‐14	7 ± 3, 8, 3‐12	7 ± 3, 8, 3‐14	4 ± 2, 4, 3‐7
Medical history at the time of injury, *n* (%)				
Prior brain disease	9 (11)	5 (11)	2 (7)	2 (33)
Diabetes mellitus	9 (11)	5 (11)	4 (14)	‐
Cardiovascular diseases	34 (43)	22 (50)	10 (35)	2 (33)
Anticoagulation treatment	27 (34)	15 (34)	11 (38)	1 (17)
Cause of accident, *n* (%)				
Driver/passenger of vehicle^b^	16 (20)	9 (21)	6 (21)	1 (17)
Pedestrian hit by vehicle	3 (4)	1 (2)	2 (7)	‐
Cyclist hit by vehicle	5 (6)	2 (5)	3 (10)	‐
Fall accident	54 (68)	31 (71)	18 (62)	5 (83)
Other^c^	1 (1)	1 (2)	‐	‐
Dominant finding on first computed tomography scan, *n* (%)				
Acute subdural hematoma	32 (41)	18 (41)	11 (38)	3 (50)
Contusions	16 (20)	8 (18)	8 (28)	‐
Traumatic subarachnoid hemorrhage	12 (15)	6 (14)	5 (17)	1 (17)
Mixed injuries	10 (13)	4 (9)	4 (14)	2 (33)
Open/closed impression fracture	4 (5)	3 (7)	1 (3)	‐
Epidural hematoma	3 (4)	3 (7)	‐	‐
Normal/other	2 (3)	2 (5)	‐	‐
Rehabilitation, *n* (%)^d^, ^e^	62 (79)	31 (71)	26 (90)	5 (83)
Need of assistance^f^, *n* (%)	37 (47)	19 (43)	13 (45)	5 (83)
Use of walking device, *n* (%)	26 (33)	14 (32)	10 (35)	2 (33)
Use of wheelchair, *n* (%)	19 (24)	9 (20)	6 (21)	4 (67)
Oropharyngeal dysphagia, *n* (%)^g^	8 (10)	6 (14)	‐	2 (33)
Impaired sense of smell and/or taste, *n* (%)^h^	21 (27)	10 (23)	11 (38)	‐
Marital status				
Living alone	22 (28)	12 (27)	8 (28)	2 (33)
Cohabitant	48 (61)	28 (64)	16 (55)	4 (67)
Living‐apart together relationship	9 (11)	4 (9)	5 (17)	‐
Vocational situation				
Old age retirement	74 (94)	40 (91)	28 (97)	6 (100)
Working full time/part time	5 (6)	4 (9)	1 (3)	‐

Abbreviations: RLS 85, Reaction Level Scale; TBI, traumatic brain injury.

^a^ Injury severity was classified as level of consciousness at the injury: Mild TBI = RLS 85 1‐3a; Moderate TBI = RLS 85 3b‐4; Severe TBI = RLS 85 5‐8.

^b^ Vehicle = car/motorcycle/moped/bicycle/snow scooter/four‐wheeler/other.

^c^ Hit the head during a sudden rise from sitting to standing.

^d^ Unknown/not assessable, *n* = 2.

^e^ Rehabilitation was provided by the municipality/primary health care and/or hospital/community based rehabilitation services.

^f^ Need of assistance is defined as personal assistance/home‐help service provided by the municipality/dependent on next‐of‐kin/significant other/help with cleaning, household, maintenance by private home care service or a combination of these.

^g^ Unknown/not assessable, *n* = 4.

^h^ Unknown/not assessable, *n* = 5.

The mean age at long‐term follow‐up was 76 years (SD ± 5) and the median 75 years (range 64–91). The mean time since injury was 7 years (SD ±3) and the median 7 years (range 3–14 years) for all the participants. In the severe TBI group, the mean time since injury was shorter (4 years) compared to the mild and moderate groups (both 7 years). The distribution between the three TBI severity groups was: mild = 44 (56%), moderate = 29 (37%), and severe = 6 (8%). In the participants' medical history, cardiovascular diseases (*n* = 34; 43%) and anticoagulation treatment (*n* = 27; 34%) were most common. Falls (*n* = 54; 68%) and vehicle accidents (*n* = 16; 20%) were the most common causes of injury. Acute subdural hematoma (*n* = 32; 41%) was the most common finding on the first computed tomography scan. A majority (79%) had received rehabilitation. Among those with a moderate and severe injury, 90% and 83%, respectively, had received rehabilitation.

About half of the participants needed some assistance at long‐term follow‐up, one third used a walking device and one quarter a wheelchair. Oropharyngeal dysphagia and impaired sense of smell and/or taste was reported among 10% and 27%, respectively, of the participants.

Twenty‐two participants (28%) were living alone at follow‐up; 13 (17%) were single both at follow‐up and at the time of injury, 6 (8%) had become widowed from the time of injury to follow‐up, and 1 male participant was single at follow‐up but had a partner at the time of injury. Fifty‐seven participants (72%) were in a relationship at follow‐up; of these, 54 (70%) were in a relationship with the same person as at the time of injury. One female participant was in a relationship at follow‐up but was widowed at the time of injury. Two of the participants were unable to give information about their marital status at the time of injury; these two participants were single at the time of follow‐up.

Seventy‐four of the participants (94%) were retired. Five participants (6%) were productive in some form at the time of follow‐up; their professional activity ranged from 2 hours per week to full‐time work.

### Changes in overall outcome

Data on GOSE at 6 months post injury and at the long‐term follow‐up for 78 participants are presented in Table 3 (one participant was excluded because of missing data at 6 months post injury; this participant was scored GOSE Lower Good Recovery at follow‐up). Thirty‐one participants (40%) had the same GOSE score at both assessments, 17 participants (22%) had a higher GOSE score at the long‐term follow‐up and 30 participants (38%) had a lower GOSE score. No participant was scored as vegetative at 6 months post injury or at the long‐term follow‐up. For the participants with a higher GOSE at the long‐term follow‐up, 11 (65%) had effects of head injury as the most important factor in outcome. For the participants with a lower GOSE at the long‐term follow‐up, 13 (43%) had the head injury as the most important factor in outcome.

**TABLE 3:** Glasgow Outcome Scale Extended for 78 participants^a^ in the Uppsala Long‐term outcome in Older adults with Traumatic brain injury Study (U‐LOTS) from 6 months post injury to the long‐term follow‐up after 3–14 years.

	GOSE at long‐term follow‐up						
	Vegetative state	Lower severe disability	Upper severe disability	Lower moderate disability	Upper moderate disability	Lower good recovery	Upper good recovery
GOSE 6 months post injury							
Vegetative state	‐	‐	‐	‐	‐	‐	‐
Lower severe disability	‐	**11**	3	‐	‐	‐	‐
Upper severe disability	‐	2	**4**	2	1	2	‐
Lower moderate disability	‐	1	1	‐	1	1	‐
Upper moderate disability	‐	2	‐	1	**1**	4	‐
Lower good recovery	‐	2	‐	1	1	**11**	3
Upper good recovery	‐	1	‐	2	5	11	**4**

*Note*: Bold values represent the same GOSE score at 6 months post injury and at the long‐term follow‐up.

Abbreviation: GOSE, Glasgow Outcome Scale Extended.

^a^ One participant was excluded because of missing GOSE data at 6 months post injury; this participant was scored GOSE Lower Good Recovery at the long‐term follow‐up.

### Functioning and disability at the long‐term follow‐up

In Table 4, data are presented on FIM Motor, FIM Cognition, and MPAI‐4 at the long‐term follow‐up and the proportion of participants who had reached the minimum score (% floor) and the maximum score (% ceiling) for each of the outcome measures. FIM Motor and FIM Cognition scores for participants that had a mild or moderate TBI were generally high with several participants (15% and 33%, respectively) reaching the maximum score (ie, ceiling effect). The participants with severe TBI had lower FIM Motor and FIM cognition scores with a high proportion (50% and 33%, respectively) of participants dependent on total assistance (ie, floor effect).

**TABLE 4:** Functional Independence Measure and Mayo‐Portland Adaptability Inventory‐4^a^ for 79 participants in the Uppsala Long‐term outcome in Older adults with Traumatic brain injury Study (U‐LOTS) at the long‐term follow‐up 3–14 years post injury.

	Mild TBI^b^ (*n* = 44)					Moderate TBI^b^ (*n* = 29)					Severe TBI^b^ (*n* = 6)				
	Mean	Median	Min‐max	% Floor^c^	% Ceiling^d^	Mean	Median	Min‐max	% Floor^c^	% Ceiling^d^	Mean	Median	Min‐max	% Floor^c^	% Ceiling^d^
FIM															
Motor	79	87	19–91	‐	11	78	88	19–91	‐	21	42	32	13–87	50	‐
Cognition	30	34	13–35	‐	36	29	32	11–35	‐	28	17	18	5–35	33	17
MPAI‐4															
Total^e^	29	26	3–56	‐	‐	26	25	‐30–56	3	‐	70	47	19–142	‐	17
Ability^f^	33	33	11–56	‐	‐	32	33	‐4–56	7	‐	68	44	27–125	‐	33
Adjustment^g^	32	31	8–54	‐	‐	28	30	‐5–60	3	‐	55	46	26–90	‐	‐
Participation^h^	39	34	23–69	7	11	38	33	23–69	3	14	61	69	30–69	‐	67

Abbreviations: FIM, Functional Independence Measure; MPAI‐4, Mayo‐Portland Adaptability‐4; RLS 85, Reaction Level Scale; TBI, traumatic brain injury.

^a^ Reference table 2015 National OutcomeInfo Sample.

^b^ Injury severity was classified as level of consciousness at the injury: Mild TBI = RLS 85 1‐3a; Moderate TBI = RLS 85 3b‐4; Severe TBI = RLS 85 5‐8.

^c^ % Floor represents the relative number (%) of participants that reached the minimum score at the time of assessment. For FIM, minimum score corresponds to total assistance. For MPAI‐4, minimum score corresponds to relatively good outcome.

^d^ % Ceiling represents the relative number (%) of participants that reached the maximum score at the time of assessment. For FIM, maximum score corresponds to complete independence. For MPAI‐4, maximum score corresponds to severe limitations.

^e^ MPAI‐4 T‐scores (min‐max): Total (‐30–142).

^f^ MPAI‐4 T‐scores (min‐max): Ability (‐4–125).

^g^ MPAI‐4 T‐scores (min‐max): Adjustment (‐5–114).

^h^ MPAI‐4 T‐scores (min‐max): Participation (23–69).

Based on the MPAI‐4 total T‐scores, 45 participants (57%) were considered to have a relatively good outcome, 8 (10%) mild limitations, 18 (23%) in the mild to moderate range, 6 (8%) in the moderate to severe range, and 2 (3%) severe limitations. MPAI‐4 scores for participants that had a mild or moderate TBI were generally low indicating relatively good outcome, whereas the participants with a severe TBI had higher MPAI‐4 scores indicating severe limitations. In comparison with FIM, a lower number of participants with mild or moderate TBI reached minimum score (1%), corresponding to a ceiling effect in the FIM. For two of the participants who were considered minimally conscious state, 4 was given on MPAI‐4 items to which they could not respond.

### Associations with the outcome measures

The associations (Spearman's correlation coefficients [ρ]) between GOSE, FIM, and MPAI‐4 and gender, age at injury, time since injury, and injury severity, respectively, are presented in Table 5. Injury severity was significantly correlated with the GOSE (*p* = .026), the FIM Cognition (*p* < .001), and FIM Motor (*p* = .033) scores, and the MPAI‐4 Ability scores (*p* = .038). There was no significant correlation between outcome and gender, age at injury, and time since injury at the long‐term follow‐up.

**TABLE 5:** Correlations between gender, age at injury, time since injury, and injury severity for 79 participants in the Uppsala Long‐term outcome in Older adults with Traumatic brain injury Study (U‐LOTS).

	Gender	Age at injury	Time since injury	Injury severity^a^
GOSE	0.051 (.65)	−0.12 (.29)	−0.096 (.40)	−0.25 (**.026**)
FIM				
Cognition	−0.17 (.13)	−0.099 (.39)	−0.089 (.44)	−0.36 **(<.001)**
Motor	0.019 (.87)	−0.16 (.17)	−0.11 (.33)	−0.24 (**.033**)
MPAI‐4^b^				
Total	0.035 (.76)	0.11 (.35)	0.087 (.45)	0.21 (.067)
Ability	0.062 (.59)	0.09 (.41)	0.056 (.62)	0.23 **(.038)**
Adjustment	0.001 (>.99)	0.062 (.59)	0.076 (.50)	0.14 (.23)
Participation	0.008 (.95)	0.19 (.091)	0.047 (.68)	0.22 (.050)

*Note*: The correlation between the variables were analyzed with the Spearman rank correlation coefficient, ρ. Corresponding *p* values are given within brackets. Bold *p* values within brackets represent statistical significance.

Abbreviations: FIM, Functional Independence Measure; GOSE, Glasgow Outcome Scale Extended; MPAI‐4, Mayo‐Portland Adaptability Inventory‐4; RLS 85, Reaction Level Scale; TBI, traumatic brain injury.

^a^ Injury severity was classified as level of consciousness at the injury: Mild TBI = RLS 85 1‐3a; Moderate TBI = RLS 85 3b‐4; Severe TBI = RLS 85 5‐8.

^b^ MPAI‐4 T‐scores are based on reference table 2015 National OutcomeInfo Sample.

## DISCUSSION

This cross‐sectional study has assessed the overall outcome, long‐term functioning, and disability of 79 surviving individuals who were 60 years or older when they sustained a TBI that required NIC. To the best of our knowledge, this is the largest study that has assessed long‐term follow‐up of a total cohort of older people surviving a TBI. The main findings were that falls and acute subdural hematoma were the most common cause and injury (68% and 41%, respectively). For many of these participants (40%) the GOSE scores did not change between 6 months post injury until long‐term follow‐up, and a majority (57%) had a relatively good outcome as assessed with the MPAI‐4. The GOSE, FIM, and MPAI‐4 Ability scores were significantly correlated with injury severity. The marital status remained unchanged for 70% of the participants.

Almost 50% of the potential participants in the UTBI register were deceased in the period between discharge from the NIC unit and the invitation to participate in the U‐LOTS. Previous studies have shown an increased risk of death, both in the postacute phase and many years after TBI compared to the general population.^39^, ^40^, ^41^ However, the reason for the death or when it had occurred remains unknown in our study population, and the ethical approval limited us in obtaining this information.

The response rate was 57% and despite several attempts to reach the nonresponders we did not succeed. Common TBI sequelae such as neurobehavioral changes, cognitive impairment, and fatigue might have contributed to difficulties in responding to the invitation.^42^, ^43^, ^44^ Overall, though, there was no statistical difference between participants and nonparticipants with regard to gender, age, age at injury, and injury severity. Men were in a majority (65%) among the participants, which is similar to many other TBI studies^45^, ^46^, ^47^ and also similar to those that have specifically looked at older adults who have sustained a TBI.^48^, ^49^ Taken together, we believe the participants in U‐LOTS are representative of the population with TBI regarding gender, age, age at injury, and injury severity.

The predominance of falls resulting in a mild TBI with an acute subdural hematoma is in agreement with other studies.^20^, ^50^, ^51^, ^52^, ^53^ The concomitant occurrence of cardiovascular diseases and anticoagulation treatment among older adults is also well known.^3^, ^9^, ^14^, ^20^, ^54^ Together, this can partly explain the high predominance of intracranial injuries and subdural hematomas in this population. Aging is associated with intracranial changes such as brain atrophy and fragile bridging veins, and older people are especially susceptible due to factors such as poor balance, which are likely to increase their vulnerability. The results reiterate that fall prevention is of the highest priority to reduce the incidence of TBI among older people.

The high degree of marital stability, where the majority of participants had been in a relationship for many years prior to the TBI and remained together with the same partner after injury, is consistent with other studies.^55^ Factors such as older age at injury and female gender are known to be associated with marital stability after TBI.^55^ The major reason for a change in marital status among our participants was death of their partner, and being a widow/er is to be expected in a population of older adults. Return to work was not an important issue because >90% of the participants had reached retirement age several years before the long‐term follow‐up. It can be a strength for older people with a TBI that the social situation is more stable with long well‐established relationships that do not change after a TBI. Also, not having to return to work may be advantageous, which can be a challenge for many younger individuals who sustain a TBI.^56^, ^57^

Several participants reported that they had impaired sense of taste and/or smell after the TBI and that it had an impact on their everyday life and participation in leisure activities. Previous studies have reported impairments of smell and taste after TBI but factors such as age and gender should also be considered as contributing. Post‐TBI olfactory dysfunction might lower quality of life^58^, ^59^ and it is therefore important to consider this among older people who have sustained a TBI.

Most of the participants who were in need of assistance received it through the municipality and in some cases combined with support from relatives. There were only a few participants who received all assistance by a significant other. In Sweden, there is a well‐established municipal system for providing help in everyday life. This means that people are not dependent on relatives to assist them in everyday life, which, in turn, may also contribute to the high degree of marital stability. Use of walking device or wheelchair is also expected to be more common among older adults and especially following a TBI.

Overall outcome as assessed with GOSE at 6 months post injury and at the long‐term follow‐up revealed that many of the participants had the same score, whereas a smaller proportion either improved over time or deteriorated. This indicates that many older adults can maintain their improvement post injury or even further improve. For a majority of those who deteriorated with regard to GOSE, the effects of illness/injury to another part of the body or combined with the effects of the TBI were reported as the most important factor for outcome. Comorbidities, including preexisting disorders that might worsen after TBI as well as new conditions (such as depression, pain, and cognitive impairment), could have an adverse effect on long‐term outcome after TBI. However, the cross‐sectional study design did not allow any further insight into the changes in GOSE. Development of comorbidity measures adapted to TBI would improve the risk evaluation and postinjury care among a population of older adults.^60^, ^61^

The FIM and MPAI‐4 indicated a relatively high level of functioning for the participants with mild and moderate TBI and, as expected, a lower level for those with severe TBI. The differences in outcome with a lower proportion of ceiling and floor effect for the MPAI‐4 may be related to the fact that the outcome measures investigate different aspects of disability following a TBI. For the FIM there is a well‐known ceiling effect, which means that the full extent of the functional outcome after TBI may not be captured using this outcome measure.^62^ The MPAI‐4 examines a much broader spectrum of common limitations after TBI than the FIM, and the differences seen in this study confirm previous findings on outcome after TBI.^63^

The significant associations between the outcome measures used and injury severity indicate that it was the most important factor for long‐term outcome. This is in line with previous studies of mixed populations also showing that injury severity is an important factor for overall outcome. Also, it is in agreement with studies specifically of older adults reporting less favorable outcomes due to increased injury severity.^64^ When interpreting the results in our study, it must be taken into account that the number of participants with severe TBI was small. The difference in average time since injury in this group compared to the mild and moderate groups should also be noted as both improvements and deteriorations may be possible over time.

The use of GCS and RLS as indicators of injury severity have well‐known limitations and measures of posttraumatic amnesia may be better in predicting outcome.^65^

The majority of participants (79%) had received rehabilitation and this is in accordance with the health care system in Sweden. Interventions are determined based on the needs and therefore there was a wide variation and different providers. These interventions may have enhanced recovery, but knowledge about this is beyond the scope of this study.

Our results have some clinical implications. In the management of TBI it is important to identify subgroups of older people most likely to achieve full recovery. Because the long‐term outcome of mild and moderate TBI is very similar to that of younger people with TBI,^66^ it is of importance that older people with TBI are assessed, treated, and rehabilitated based on injury severity and functional level instead of strict age limits.^67^ Further studies are also needed to increase our knowledge of which factors that contribute to long‐term outcome in older people following a TBI.

### Strengths and limitations

A strength of the study is the use of an established register to recruit participants and obtain baseline information. We were therefore able to invite everyone who met the inclusion criteria, which, in turn, means that this study comprises an entire population of people with TBI treated at a NIC over an extended period of time. The same researcher (CvS), with extensive experience from TBI rehabilitation, pursued all assessments, and 89% of the assessments were performed face to face with standardized interviews and internationally established TBI outcome measures. Thereby, a detailed description of the participants' overall outcome, functioning, and disability at follow‐up were obtained. Remaining data in the U‐LOTS and forthcoming studies will provide new insights into factors of importance for healthy aging among older people who have sustained a TBI.

A limitation is that the sample comprised older adults who had been admitted to the NIC unit and not the entire population of older adults with TBI in the catchment area. This can cause a selection bias as patients with better prognosis are more often transferred to a NIC unit. It was not possible to obtain data, including cause of death, on the 170 potential participants who were deceased at the time of invitation. The low response rate could also have contributed to a selection bias, as people with better long‐term outcome, milder TBI, or strong social support might be more willing to agree to participate than those with severe TBI and absence of a social network. The small sample size, especially the small number of severely injured people, limits the ability to make more detailed inferences.

### Conclusions

The global outcome in this study cohort of older adults surviving a TBI was relatively favorable, and the results from the long‐term follow‐up showed that most participants' outcome remained unchanged whereas some participants improved and some deteriorated. A major factor that determined outcome among the participants was injury severity, where those with a mild TBI had the potential to recover quite well. Despite a life‐changing injury and lifelong disability, the participants' marital status was stable.

## FUNDING INFOMATION

This study was supported by grants from the Norrbacka Eugenia Foundation Sweden, Research funds at the Uppsala University Hospital, Fonden för rehabilitering och medicinsk forskning, Sweden, and The Brain Injury Association, Hjärnkraft, Sweden.

## DISCLOSURE

None.
